# Crtc1 deficiency protects against sepsis-associated acute lung injury through activating akt signaling pathway

**DOI:** 10.1186/s12950-024-00385-y

**Published:** 2024-04-22

**Authors:** Meng Chen, Jian Lv, Ningning Guo, Tuo Ji, Yu Fang, Zhihua Wang, Xianghu He

**Affiliations:** 1 Department of Anesthesiology, Zhongnan Hospital of Wuhan University, 430071 Wuhan, Hubei China; 2 Department of Anesthesiology, Maternal and Child Health Hospital of Hubei Province, 745 Wuluo Road, 430070 Wuhan, Hubei China; 3 Shenzhen Key Laboratory of Cardiovascular Disease, Fuwai Hospital Chinese Academy of Medical Sciences, 518057 Shenzhen, China; 4 State Key Laboratory of Cardiovascular Disease, Fuwai Hospital, National Center for Cardiovascular Diseases, Chinese Academy of Medical Sciences and Peking Union Medical College, 100037 Beijing, China; 5 Department of Anesthesiology, School and Hospital of Stomatology, Wuhan University, 430079 Wuhan, China

**Keywords:** Crtc1, Acute lung injury, Sepsis, Inflammation, Apoptosis

## Abstract

**Background:**

Interplay between systemic inflammation and programmed cell death contributes to the pathogenesis of acute lung injury (ALI). cAMP-regulated transcriptional coactivator 1 (CRTC1) has been involved in the normal function of the pulmonary system, but its role in ALI remains unclear.

**Methods and results:**

We generated a *Crtc1* knockout (KO; *Crtc1*^−/−^) mouse line. Sepsis-induced ALI was established by cecal ligation and puncture (CLP) for 24 h. The data showed that *Ctrc1* KO substantially ameliorated CLP-induced ALI phenotypes, including improved lung structure destruction, reduced pulmonary vascular permeability, diminished levels of proinflammatory cytokines and chemokines, compared with the wildtype mice. Consistently, in lipopolysaccharide (LPS)-treated RAW264.7 cells, *Crtc1* knockdown significantly inhibited the expression of inflammatory effectors, including TNF-α, IL-1β, IL-6 and CXCL1, whereas their expressions were significantly enhanced by *Crtc1* overexpression. Moreover, both *Crtc1* KO in mice and its knockdown in RAW264.7 cells dramatically reduced TUNEL-positive cells and the expression of pro-apoptotic proteins. In contrast, *Crtc1* overexpression led to an increase in the pro-apoptotic proteins and LPS-induced TUNEL-positive cells. Mechanically, we found that the phosphorylation of Akt was significantly enhanced by *Crtc1* knockout or knockdown, but suppressed by *Crtc1* overexpression. Administration of Triciribine, an Akt inhibitor, substantially blocked the protection of *Crtc1* knockdown on LPS-induced inflammation and cell death in RAW264.7 cells.

**Conclusions:**

Our study demonstrates that CRTC1 contribute to the pathological processes of inflammation and apoptosis in sepsis-induced ALI, and provides mechanistic insights into the molecular function of CRTC1 in the lung. Targeting CRTC1 would be a promising strategy to treat sepsis-induced ALI in clinic.

**Supplementary Information:**

The online version contains supplementary material available at 10.1186/s12950-024-00385-y.

## Introduction

Sepsis is a serious illness caused by infection with high incidence and fatality rate, leading to tissue damage and organ failure, particularly acute respiratory distress syndrome (ARDS) / acute lung injury (ALI) [1]. ALI is characterized by sudden onset, widespread lung damage, disrupted gas exchange, and extensive infiltration of neutrophils, macrophages, erythrocytes in the alveoli, as well as necrosis of lung endothelial and epithelial cells [2, 3]. Alveolar macrophages (AMs), as sentinel phagocytes of the pulmonary innate immune system, are closely associated with the pathogenesis of ALI [4].

The pathogenesis of sepsis mainly includes toxins released by pathogens, inflammatory factors, neutrophil activation, and coagulation dysfunction. Overproduction of inflammatory factors and cell death contribute to the development of damage, whereas inhibiting inflammation and cell death can significantly protect against ALI/ARDS and improve outcomes [5]. How the inflammation and apoptosis pathways are coordinated during the pathogenesis of ALI remains largely unknown.

cAMP regulated transcriptional coactivators (CRTCs) are a family of conserved transcriptional coactivators that sense cellular signaling pathways and govern the expression of specific genes. As the first identified member of the CRTCs family, CRTC1 has been involved in eating behavior [6], energy balance [7], cancer [8, 9], and degenerative diseases [10]. CRTC1 has been closely linked to the development of lung-related illnesses. A clinical trial revealed that inhibiting CRTC1 can enhance immune function and lower respiratory infections in older individuals [11]. Moreover, an examination of gene expression data showed that CRTC1 and its target gene, SEC14L3, were differentially expressed in individuals with asthma [12], suggesting that CRTC1 may play a role in regulating conditions such as bronchial asthma or other lung diseases. However, there have been no reports on the role and potential mechanisms of CRTC1 in ALI.

Here we generated a *Crtc1* knockout (KO; *Crtc1*^−/−^) mouse line and found that *Crtc1* deficiency significantly ameliorated cecal ligation and puncture (CLP)-induced ALI inflammation and cell apoptosis. The role of CRTC1 was further validated in lipopolysaccharide (LPS)-treated RAW264.7 cells using knockdown and overexpression strategies. Triciribine, an Akt inhibitor, significantly blocked the protection against LPS-induced pathologies by *Crtc1* knockdown. Our findings reveal a detrimental role of CRTC1 in sepsis-induced ALI, and provide a novel and promising target to treat infection-associated ALI in clinic.

## Materials and methods

### Animals

The *Crtc1* KO (*Crtc1*^−/−^) male mice and littermate wild-type (*Crtc1*^+/+^) male mice (8–9 weeks) were maintained and bred as we previously reported [13].

### Surgical procedure and sample collection

Polymicrobial sepsis was induced by CLP surgery to establish the sepsis-induced ALI model as reported previously [14]. Before the surgery operation, the mice were fasted for 12 h with free access to water. Under anesthesia with pentobarbital (50 mg / kg, i.p), a 1 cm midline incision was made along the linea alba after the opening of the abdominal, allowing the cecum to be exposed. The cecum was ligated with a nonabsorbable suture at 50% from the colon root and punctured twice by a 22-G needle, and a small amount of the intestinal content was forced out to induce sepsis. Finally, the cecum was reset and the abdominal incision was closed. For the sham group, animals underwent abdominal incision and intestinal mobilization with neither ligation nor puncture. All animals were harvested with an overdose of pentobarbital, bronchoalveolar lavage fluid (BALF), serum and lung tissues were collected 24 h after the surgery. The left lobe of the lung was used for subsequent histological analyses, and the right lobes were used for the evaluation of biochemical parameters.

### ELISA in BALF

BALF was harvested via injection and retraction of 1.0mL of precooled 0.9% NS for three times through the trachea. The collected BALF samples were then centrifuged. The cell-free supernatant was sub-packed and stored at -80℃ for protein and cytokine testing. BALF was centrifuged at 12,000 rpm for 15 min, and the supernatant fractions were aliquoted and stored at -80 °C. The concentration of inflammatory protein IL-6 (MKC00B) or TNF-α (MM200) in the supernatants were measured using ELISA kits (R&D Systems), according to the manufacturer’s instructions.

### Lung histology

Mouse left lung samples from all groups were fixed in 4% paraformaldehyde and then embedded in paraffin. Tissue blocks were sliced at 5 μm and stained with hematoxylin and eosin (H&E). Lung injury score was measured by two pathologists blinded to the experiment. The histological injury was graded using the method we previously described [15]: grade 0, no diagnostic change; grade 1, mild neutrophil infiltration and mild to moderate interstitial congestion; grade 2, moderate neutrophil infiltration, perivascular edema and partial destruction of pulmonary architecture; and grade 3, dense infiltration of neutrophils and complete destruction of pulmonary architecture.

### Evans blue

To further test the lung capillary leakage, Evans blue dye (20 mg/kg) was injected through the tail vein 30 min before mice were sacrificed. Then, the EBD in the pulmonary circulatory system was rinsed with ice-cold PBS. Lung tissues were harvested and homogenized in 1mL of formamide per 0.1 mg of tissue and incubated at 37℃ for 12 h to extract EBD. Then, the lysates were centrifuged at 5,000 g for 30 min. The EB concentration of the tissues was examined by measuring the optical density of the supernatant at 620 nm. The content of EBD in lung homogenate was calculated based on the standard curve and was expressed as mg of EBD / g of lung tissue.

### Cell culture and treatment

Murine RAW264.7 cells were purchased from the cell bank of Chinese Academy of Sciences (Shanghai, China). The cells were maintained in DMEM culture medium supplemented with 10% FBS (Gibco, Carlsbad, USA), followed by culture in a 5% CO2 / 95% air humidified atmosphere at 37 °C. DNA transfection and interference RNA using Lipofectamine2000 (Invitrogen, Carlsbad, CA) according to the manufacturer’s instructions. Transfection was performed with Opti-MEM (Gibco, Carlsbad, USA) instead of culture medium. After 6–8 h, the Opti-MEM was replaced with standard DMEM culture medium as described above. When the cells reached 75% confluence, they were cultured with culture medium with LPS (1 µg/mL) or PBS for 4 h and then collected for qRT-PCR, immunofluorescence, and western blot assays.

To explore the role of the Akt pathway in ALI, the PI3K inhibitor Triciribine was dissolved in dimethyl sulfoxide (DMSO) (10 μm), then added to cell cultures for 24 h before subjecting them to LPS.

### TUNEL staining

Apoptosis was detected by Terminal deoxynucleotidyl transferase (TdT) dUTP Nick-End Labeling (TUNEL) assay kit (Roche, Boehringer Mannheim, Germany). Lung tissue slices and the cells were immersed with 4% paraformaldehyde for 15 min and then cleaned with PBS for three times. Fixed slices were incubated with TUNEL reaction solution for 60 min at 37 °C in dark, followed by cleaning three times. Next, slices were soaked with DAPI solution for 7 min. The total cells and apoptotic cells were observed and determined under a fluorescence microscope (Olympus, Tokyo, Japan). Apoptosis index = number of TUNEL-positive cells / total cells.

### qRTPCR

Lung tissues and RAW264.7 cells were homogenized in RNAiso plus (Takara, Shiga, Japan) lysis buffer, and total RNA was extracted. RNAs were reverse-transcribed using reverse transcription reagents (Thermo Scientific, USA). qRT-PCR was performed using SYBR Green Master Mix on LightCycler 480 sequence-detector system. All the primers in this study were obtained from Sangon Biotech (Shanghai, China). β-actin or GAPDH was used as the internal control. The comparative Ct method (2^− ΔΔCt^) was utilized to analyze data.

### Western blot assay

Lung tissues and cells were homogenized and lysed in RIPA buffer (Beyotime, Jiangsu, China) containing protease and phosphatase inhibitors (Roche, Mannheim, Germany). Total protein quantified by a BCA kit (Thermo Fisher Scientific, USA). The proteins were separated on 10% SDS-PAGE and then transferred onto PVDF membranes (Thermo Fisher Scientific). Subsequently, the membrane was blocked with 5% fat-free milk. After that, the bands were incubated with primary antibodies at 4 °C overnight on a shaker, and then with secondary antibodies at room temperature for 1 h. After washing three times with TBST, the blots were visualized by ECL (Beyotime, Jiangsu, China) and band densities were quantitatively analyzed using ImageJ software.

### Statistical analysis

All statistical analyses were performed by GraphPad Prism 8.0 (GraphPad Prism Software, USA). Student’s t-test was used to evaluate the differences between two groups for normally distributed variables with equal variance. For normally distributed variables with equal variance, differences among three groups were compared by one-way analysis of variance (ANOVA), followed by post hoc Turkey’s tests. All values were represented as mean ± SD. The significant difference was set at *P* < 0.05.

## Results

### Crtc1 deficiency ameliorated CLP-induced ALI

**Fig. 1:**
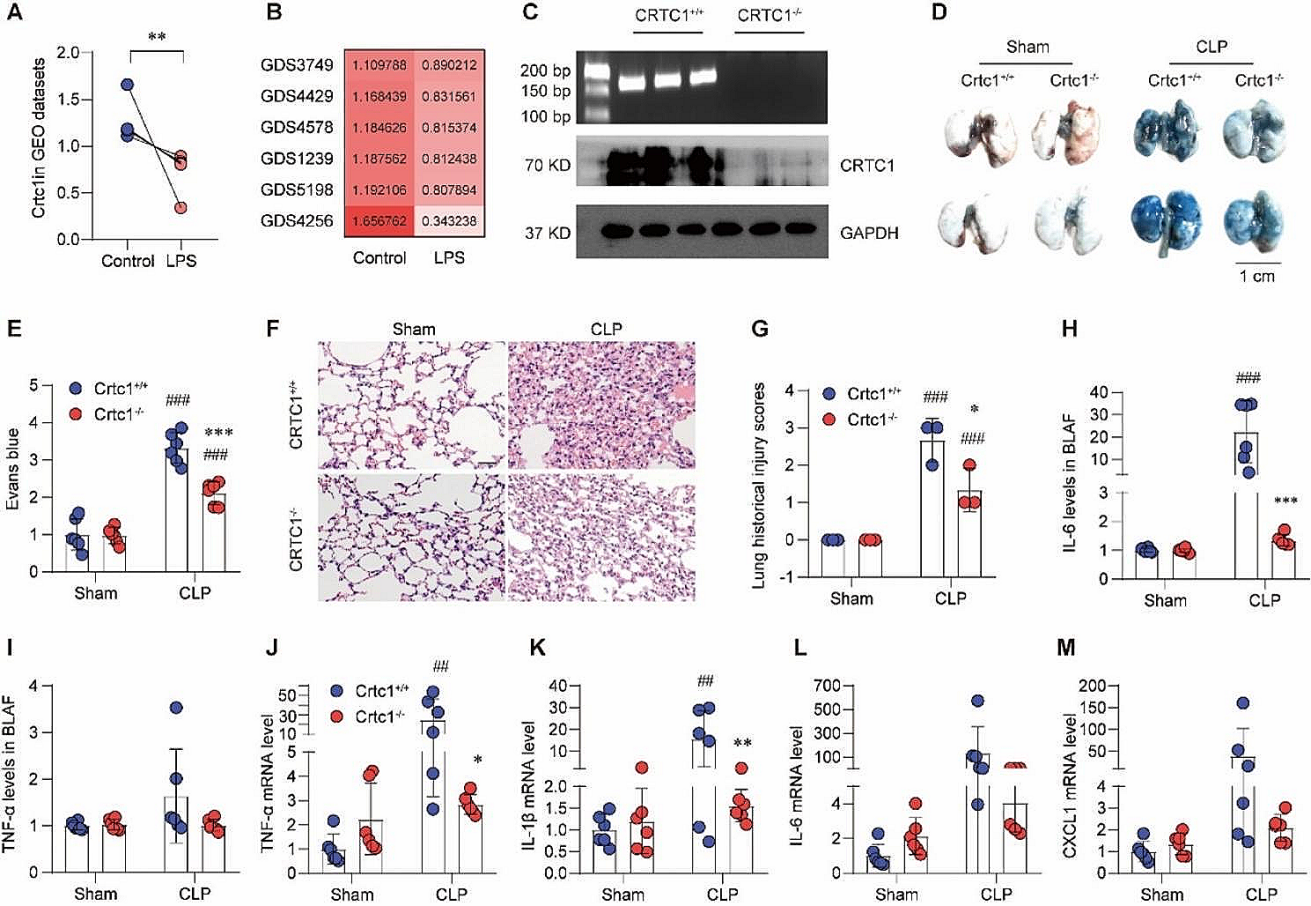
Crtc1 deficiency ameliorated CLP-induced ALI. (**A, B**) Scatter plot (**A**) and heat map (B) of Crtc1 mRNA changes in LPS-induced inflammation models from different datasets in the GEO database. (**C**) Crtc1 knockout was validated through DNA genotyping (upper) and Western blot (lower) in lung tissue. *n* = 3. (**D, E**) Representative images (**D**) and quantitation (**E**) of Evans blue albumin to evaluate pulmonary vascular permeability in Crtc1 KO mice, compared with WT mice. ^***^P < 0.001 vs. Crtc1^+/+^; ^###^*P* < 0.001 vs. Sham. *n* = 6. (**F, G**) Representative H&E staining images (**F**) and ALI scoring (**G**) in lung tissue. ^*^P < 0.05 vs. Crtc1^+/+^; ^###^*P* < 0.001 vs. Sham. *n* = 3. (H, I) Cytokine IL-6 (**H**) and TNF-α (**I**) in BALF were detected by ELISA. ^***^P < 0.001 vs. Crtc1^+/+^; ^###^*P* < 0.001 vs. Sham. *n* = 6. (**J-M**) Impact of Crtc1 deficiency on CLP-induced Cytokines including TNF-α (**J**), IL-1β (**K**), IL-6 (**L**) and CXCL11 (**M**) in lung tissue detecting by qRT-PCR. ^*^P < 0.05, ^**^P < 0.01 vs. Crtc1^+/+^; ^##^*P* < 0.01 vs. Sham. *n* = 6

We previously generated a *Crtc1*^−/−^ mouse line using CRISPR/Cas9 and found that *Crtc1* deficiency caused spontaneous obesity through regulating the PPARγ pathway [13]. Gene expression analysis found that the level of *Crtc1* mRNA was steadily down-regulated in LPS treatment in several datasets in GEO database (Fig. 1A, B). To investigate the role of CRTC1 in ALI, we subjected the *Crtc1*^−/−^ mice (Fig. 1C), together with wildtype (WT; *Crtc1*^+/+^) littermates, to CLP surgery. The results showed that *Crtc1* deficiency significantly decreased the CLP-induced extravasation of Evans blue, compared with WT mice (Fig. 1D, E). Moreover, *Crtc1* KO ameliorated ALI pathologies, including lung septum thickness, pulmonary vascular congestion, and inflammatory cell infiltration (Fig. 1F). Consistently, ALI scoring in the *Crtc1*^−/−^ mice was significantly decreased compared with the WT mice after CLP surgery (Fig. 1G), indicating that *Crtc1* deficiency ameliorated CLP-induced ALI.

Inflammatory response in ALI involves increased production of cytokines and chemokines. ELISA assay detected significant decrease in BALF IL6 and TNFα in the *Crtc1*^−/−^ mice compared with the WT controls after CLP surgery (Fig. 1H, I). In addition, qRT-PCR showed that *Crtc1* deficiency attenuated the CLP-induced upregulation of cytokines and chemokines in the lung (Fig. 1J-M). These data suggest that CRTC1 contributes to the pathogenesis of sepsis-induced ALI.

### Crtc1 KO alleviated CLP-induced apoptosis

**Fig. 2:**
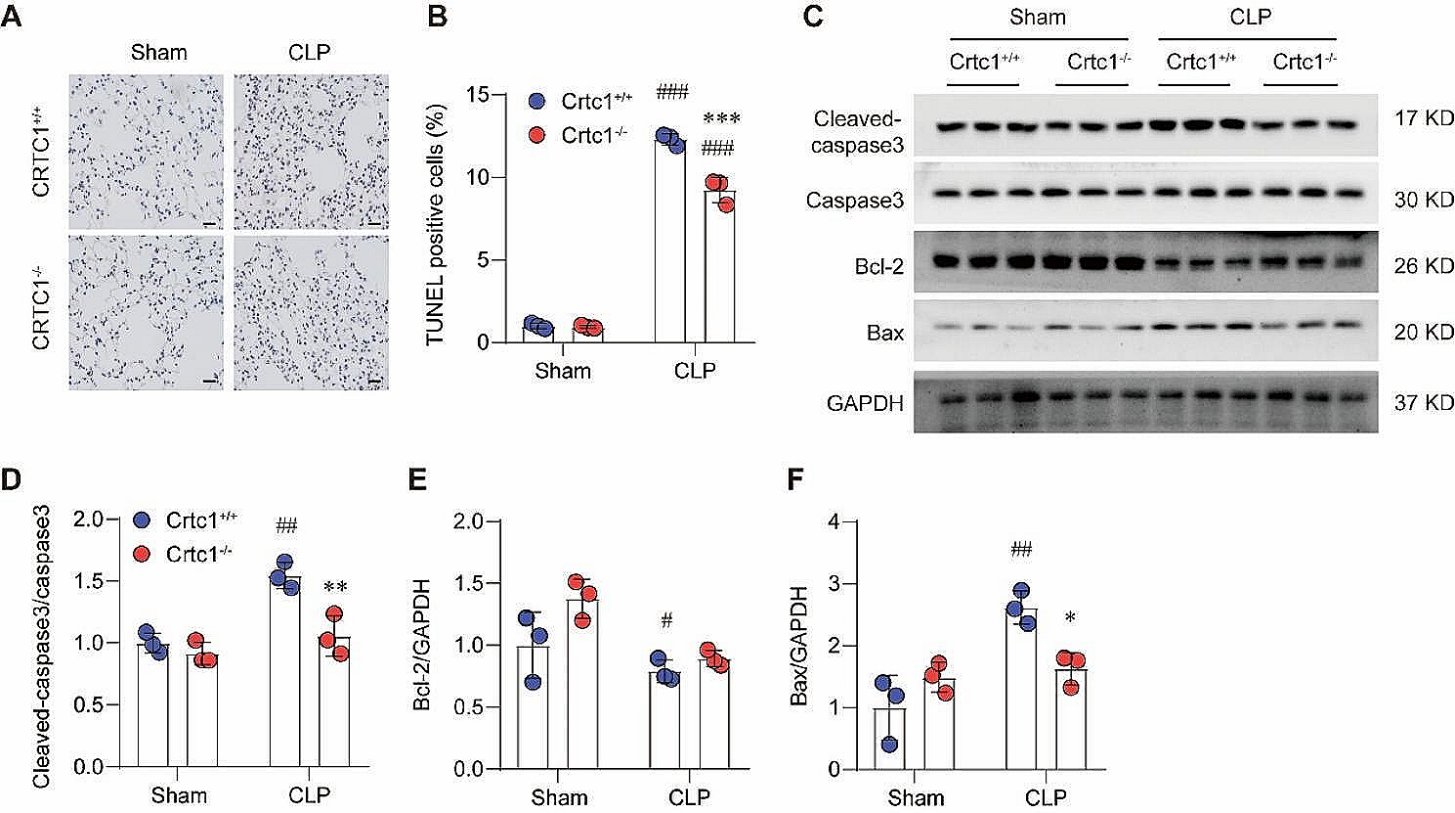
Crtc1 knockout alleviated CLP-induced apoptosis in ALI.(**A, B**) Representative TUNEL staining images (**A**) and quantification data (**B**) showing the impact of Crtc1 KO on CLP-induced apoptosis in lung tissues. ^***^P < 0.001 vs. Crtc1^+/+^; ^###^*P* < 0.001 vs. Sham. *n* = 6. (**C-F**) Western blot (**C**) and quantification (**D-F**) showing the protein level of Cleaved-caspase3/Caspase3 (**D**), Bcl-2 (**E**) and Bax (**F**) in mice lung tissues. ^*^P < 0.05, ^**^P < 0.01 vs. Crtc1^+/+^; ^#^*P* < 0.05, ^##^*P* < 0.01 vs. Sham; *n* = 3

Cell death is not only a pathological feature of ALI, but also a trigger of inflammation that might aggravate ALI pathologies [16]. We then evaluated the effect of *Crtc1* deficiency on apoptosis using TUNEL staining in CLP-induced ALI. CLP significantly increased the number of TUNEL-positive cells, which was substantially inhibited in *Crtc1*^−/−^ group (Fig. 2A, B). Consistently, the increases in cleaved-Caspase3 and Bax expression induced by CLP were significantly reversed by *Crtc1* deficiency, while the reduced level of Bcl-2 was inversely increased (Fig. 2C-F). These results suggest that *Crtc1* knockout alleviated CLP-induced apoptosis in ALI.

### Crtc1 deficiency attenuates LPS-induced inflammation and apoptosis in RAW264.7 cells

**Fig. 3:**
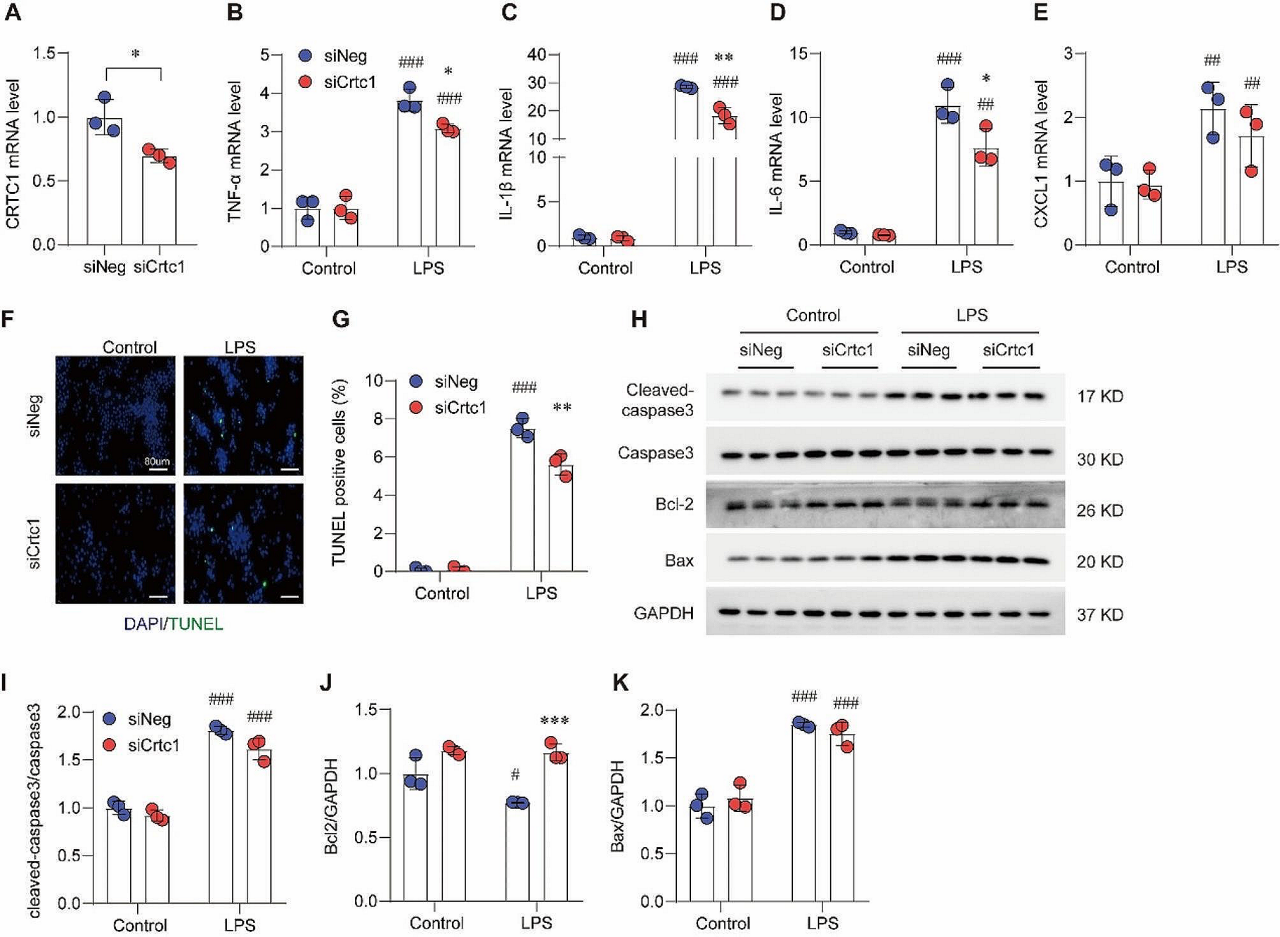
Crtc1 Silencing attenuates LPS-induced inflammation and apoptosis in RAW264.7 cells. (**A**) Validation of Crtc1 knockdown in in RAW264.7 cells treated with Crtc1-specific or negative control siRNAs using qRT-PCR. *P < 0.05; *n* = 3. (**B-E**) qRT-PCR showing the impact of Crtc1 knockdown on LPS-induced cytokines, such as TNF-α (**B**), IL-1β (**C**), IL-6 (**D**), and CXCL1 (**E**) in RAW264.7 cells. ^*^P < 0.05, ^**^P < 0.01 vs. siNeg;^##^*P* < 0.01, ^###^*P* < 0.001 vs. Control; *n* = 6. (**F**, **G**) Representative TUNEL staining images (**F**) and quantification data (**G**) showing the impact of Crtc1 knockdown on LPS-induced apoptosis in RAW264.7 cells. ^**^P < 0.01 vs. siNeg; ^###^*P* < 0.001 vs. Control; *n* = 3. (**H-K**) Western blot (**H**) and quantification (**I-K**) showing the impact of Crtc1 knockdown on LPS-induced alternations in Cleaved-caspase3/Caspase3 (**I**), Bcl-2 (**J**) and Bax (**K**) in RAW264.7 cells. ^**^P < 0.01, ^***^P < 0.001 vs. siNeg; ^#^*P* < 0.05, ^###^*P* < 0.001 vs. Control; *n* = 3

Macrophage plays a central role in the initiation, maintenance, and remission of inflammation during infection [17]. To further determine the protective effect of *Crtc1* deficiency against inflammation and apoptosis, we silenced *Crtc1* in LPS-stimulated RAW264.7 macrophages using specific siRNA (Fig. 3A). Consistent with our observation with *Crtc1* KO mice, *Crtc1* silencing significantly reduced the LPS-induced upregulation of TNF-α, IL-1β, IL-6 and CXCL1 (Fig. 3B-E). Moreover, *Crtc1* knockdown significantly inhibited the LPS-induced apoptosis, as evidenced by the decrease in TUNEL-positive cells, attenuation of cleaved-Caspase3 and Bax expressions, as well as down-regulation of Bcl-2 level induced by LPS treatment (Fig. 3F-K). These data suggest that *Crtc1* contributes to LPS-induced inflammation and apoptosis in RAW264.7 cells.

### Crtc1 overexpression increases LPS-induced inflammation and apoptosis in RAW264.7 cells

**Fig. 4:**
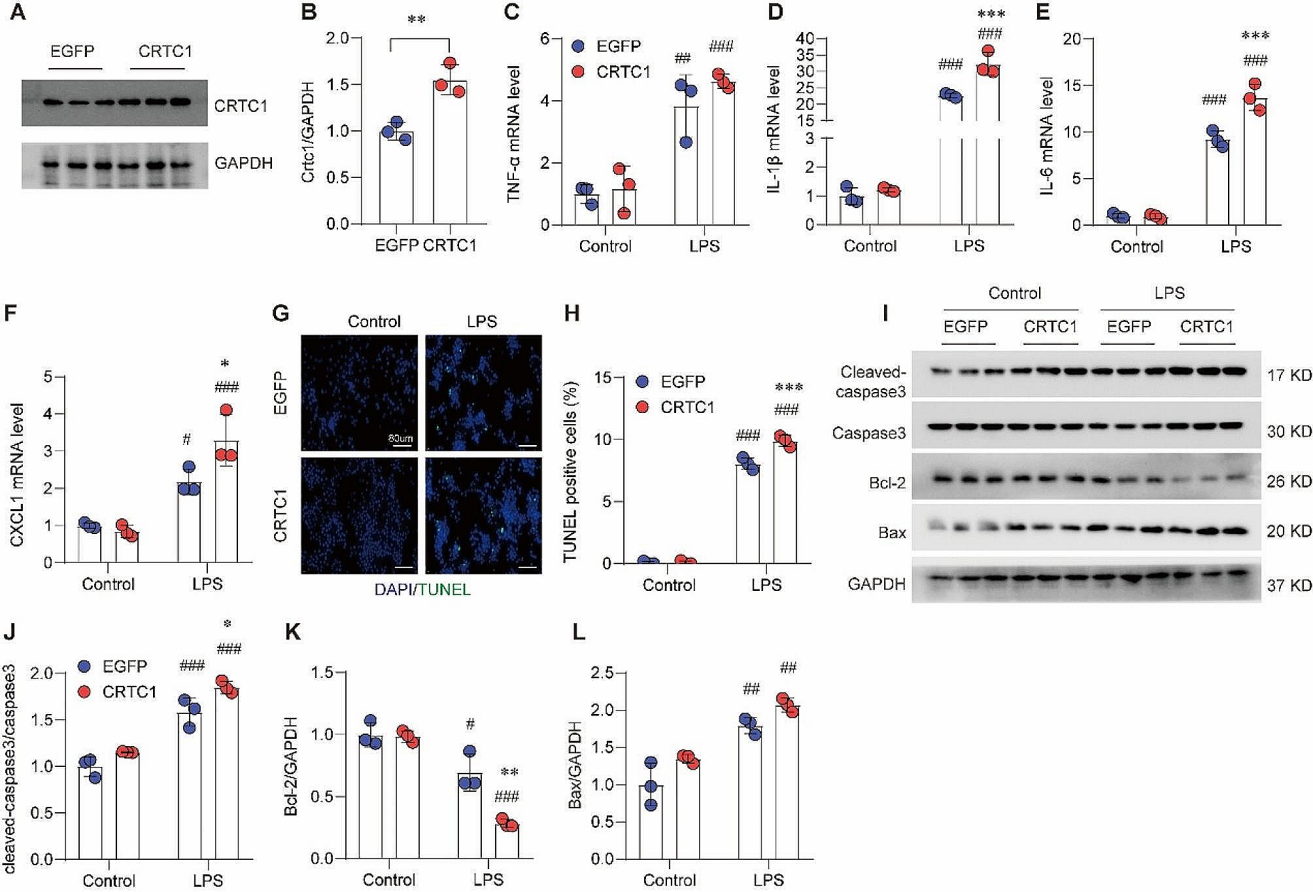
Crtc1 overexpression increases LPS-induced inflammation and apoptosis in RAW264.7 cells. (**A, B**) Western blot (**A**) and quantification (**B**) of Crtc1 overexpression in RAW264.7 cells. **P < 0.01; *n* = 3. (**C-F**) qRT-PCR showing the impact of Crtc1 overexpression on LPS-induced expression of TNF-α (**C**), IL-1β (**D**), IL-6 (**E**), and CXCL1 (**F**) in RAW264.7 cells. ^*^P < 0.05, ^***^P < 0.001 vs. EGFP;^#^*P* < 0.05, ^##^*P* < 0.01, ^###^*P* < 0.001 vs. Control; *n* = 6. (**G, H**) Representative TUNEL staining images (**G**) and quantification (H) showing the impact of Crtc1 overexpression on LPS-induced apoptosis in RAW264.7 cells. ^***^P < 0.001 vs. EGFP; ^###^*P* < 0.001 vs. Control; *n* = 3. (**I-L**) Western blot (**I**) and quantification (**J-L**) showing the impact of Crtc1 overexpression on LPS-induced alternations in Cleaved-caspase3/Caspase3 (**J**), Bcl-2 (**K**) and Bax (**L**) in RAW264.7 cells. ^*^P < 0.05, ^**^P < 0.01 vs. EGFP; ^#^*P* < 0.05, ^###^*P* < 0.001 vs. Control; *n* = 3

To further confirm the function of CRTC1 in LPS-induced ALI, we examined the impact of *Crtc1* overexpression on inflammation and apoptosis in RAW264.7 cells (Fig. 4A, B). As expected, *Crtc1* overexpression further enhanced the LPS-induced upregulation of TNF-α, IL-1β, IL-6 and CXCL1 at the mRNA level (Fig. 4C-F). *Crtc1* overexpression significantly exacerbated the LPS-induced apoptosis phenotypes, as evidenced by markedly increased TUNEL-positive cells, elevated levels of cleaved-Caspase3 and Bax, and diminished expression of Bcl-2 under LPS treatment (Fig. 4G-L). These data suggest that CRTC1 plays a detrimental role in inflammation and apoptosis in macrophages under infection.

### Akt mediates the pro-survival effects of Crtc1deficiency

**Fig. 5:**
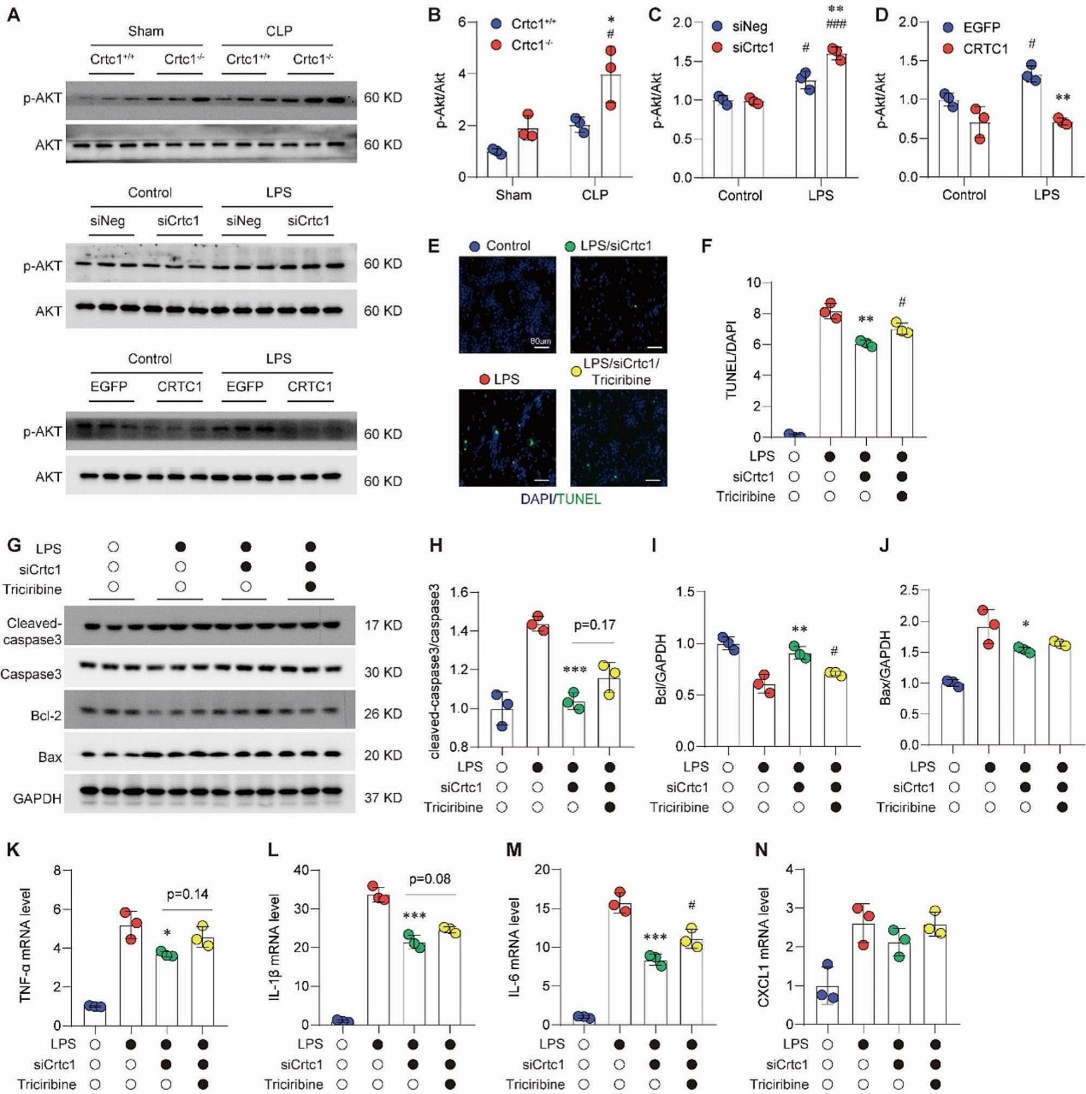
Akt signaling pathway mediates the pro-apoptosis role Crtc1 in ALI. (**A-D**) Western blot (**A**) and quantification (**B-D**) showing the role of Crtc1 gene manipulation on p-Akt/Akt. ^*^P < 0.05, ^**^P < 0.01 vs. Crtc1^+/+^; ^#^*P* < 0.05, ^###^*P* < 0.001 vs. Sham; *n* = 3. (**E, F**) Representative TUNEL staining images (**E**) and quantification (**F**) showed Triciribine (10µM) blocked the inhibition of Crtc1 knockdown on LPS-induced TUNEL-positive cells. ^**^P < 0.01 vs. LPS group; ^#^*P* < 0.05 vs. siCrtc1 + LPS group; *n* = 3. (**G-J**) Western blot (**G**) and quantification (**H-J**) showing Triciribine reversed the protective effects of Crtc1 silencing on apoptosis-associated proteins, such as Cleaved-caspase3/Caspase3 (**H**), Bcl-2 (**I**) and Bax (**J**) in RAW264.7 cells. ^*^P < 0.05, ^**^P < 0.01, ^***^P < 0.001 vs. LPS group; ^#^*P* < 0.05 vs. siCrtc1 + LPS group; *n* = 3. (**K-N**) Triciribine blocked the inhibition of Crtc1 knockdown on LPS-induced inflammation factors including TNF-α (**K**), IL-1β (**L**), IL-6 (**M**) and CXCL1 (**N**). ^*^P < 0.05, ^***^P < 0.001 vs. LPS group; ^#^*P* < 0.05 vs. siCrtc1 + LPS group; *n* = 6

Akt pathway has been involved in the maintenance of cell apoptosis during ALI [18]. Western blot analysis showed that Akt may mediate the pro-survival effects of Crtc1 (Fig. 5A-D). In detail, Crtc1 deficiency significantly promoted the phosphorylation of Akt at Ser473, compared with the WT group, with or without CLP surgery (Fig. 5A, B). Furthermore, the phosphorylation of Akt was enhanced by *Crtc1* knockdown under LPS treatment, but suppressed by *Crtc1* overexpression in both baseline and LPS treatment (Fig. 5A, C, D).

We next asked whether the Akt signaling pathway mediates the protective role of *Crtc1* deficiency against inflammation and apoptosis. Treatment with Triciribine (10 μm), an Akt inhibitor, significantly blocked the inhibition of *Crtc1* knockdown on LPS-induced TUNEL-positive cells (Fig. 5E, F). Consistently, the protective effects of *Crtc1* silencing on LPS-induced increases in cleaved caspase-3 and Bax and decrease in Bcl2 were substantially reversed by Triciribine treatment (Fig. 5G-J). Meanwhile, Triciribine largely reversed the inhibition of *Crtc1* silencing on the LPS-induced increases in TNF-α, IL-1β, IL-6 and CXCL1 (Fig. 5K-N). These data suggest that Akt signaling pathway contribute to the beneficial effects of *Crtc1* deficiency in ALI-associated inflammation and apoptosis.

## Discussion

The pathogenesis of sepsis-related acute lung injury (ALI) involves an inflammatory cascade and accumulation of apoptosis. Our study demonstrates that CRTC1 contributes to the pathogenesis of sepsis-induced ALI, while *Crtc1* deficiency protects against ALI-associated inflammation and apoptosis both in vitro and in vivo.

As a member of the CREB transcription co-factor family, CRTC1 acts as a transducer to regulate the transcriptional activity of CREB in the absence of cAMP stimulation [7]. Studies have linked CRTC1 to the progression of lung-related diseases. One clinical study showed that selectively inhibiting CRTC1 improved the immune function and reduced infection rates, particularly respiratory infection rates in elderly people [11]. Another study identified significant difference in the expression of CRTC1 and its target gene SEC14L3 in asthma, suggesting that CRTC1 plays a regulatory role in bronchial asthma or other lung diseases [12]. Our findings indicate that *Crtc1* deficiency significantly ameliorates the sepsis-induced ALI symptoms, including decreased lung structure damage, reduced pulmonary vascular permeability, and lowered the levels of pro-inflammatory cytokines and chemokines. Consistent with the in vivo observations, the inflammatory response in LPS-treated RAW264.7 cells is substantially rescued by *Crtc1* knockdown, but exacerbated by its overexpression. These data confirm an autonomic pro-inflammatory role played by CRTC1 during the pathogenesis of ALI.

Apoptosis plays a crucial role in lung injury and is thought to be closely related to the severity of lung diseases [19–21]. Martin et al. [22] showed that extensive apoptosis of pulmonary alveolar epithelial cells is responsible for the destruction of alveolar walls and the remodelling of some mesenchymal cells in ALI. TUNEL staining of impaired DNA fragments and western blot of apoptosis-associated protein are typical strategies for detecting cell apoptosis [23, 24]. Our data showed that both *Crtc1* knockout in CLP-treated mice and its knockdown in LPS-treated RAW264.7 cells dramatically decreased TUNEL-positive cells and pro-apoptotic proteins. Meanwhile, *Crtc1* overexpression in LPS-treated RAW264.7 cells caused increases in pro-apoptotic proteins and TUNEL-positive cells, suggesting a pro-apoptotic role of CRTC1 in the context of lung injury. Failure to appropriately clear the debris of the dead cells might further aggravate systemic inflammatory responses that may contribute to the severity of the ALI symptoms.

As one of the major signal transduction pathways, Protein kinase B (Akt) signaling pathway regulates various physiological activities, such as apoptosis [18], autophagy [25], and ferroptosis [26], has been proven to be a protective factor in multiple organs, such as the kidney [27], liver [28] and lung [29]. Several studies revealed Akt signaling pathway play an important role in the pulmonary inflammation and pathology of ALI through regulating cell survival and cell apoptosis. Adipose-derived mesenchymal stem cells protect lung endothelial cells from sepsis-induced lung injury through activating the PI3K/Akt axis [30]. Numerous studies have reported that the Akt signalling pathway mediates the protection against cell apoptosis in lung damage. Nrf2-/- deficiency leads to decreased Akt activation and exacerbated lung damage [29]. Lu et al. [31] discovered that activation of Akt decreased lung epithelial cell death by regulating apoptotic proteins Bax and Bcl-2. Our previous study also found that SERINC2 functions as an endogenous protector against sepsis-associated ALI through activation of the Akt pathway [32]. In addition, several Akt activators have been reported to be protective in lung damage through inhibiting cell apoptosis. Acetylvanillin reduces oxidative stress and enhances antioxidant defense by enhancing the PI3K/Akt/mTOR signaling, preventing lung injury caused by cyclophosphamide in rats [33]. Artesunate ameliorates sepsis-induced ALI by inhibiting inflammatory mediator production and apoptosis via the activation of Akt [34]. CREB-independent activation of the Akt pathway contributed to the therapeutic effects of olprinone and corforsin daropate on septic ALI through transfecting a cyclic AMP response element binding protein (CREB) decoy oligodeoxynucleotide [35]. However, our results showed that *Crtc1* knockout/knockdown significantly enhanced the phosphorylation of Akt both in vivo and in vitro, which was inversely suppressed by Crtc1 overexpression. These data suggest that the impact of CREB on Akt, either activation or inactivation, might be context-dependent. Treatment with the Akt inhibitor Triciribine substantially blocked the protective effects of Crtc1 deficiency on inflammation and cell death. These data demonstrate that the Akt signaling pathway mediates the protection of CRTC1 inhibition in ALI-associated inflammation and apoptosis.

## Conclusions

In summary, our data demonstrate that Crtc1 deficiency provided a protective effect against sepsis-associated ALI through activating Akt signaling pathway. Our findings provide novel insights into the molecular function of CRTC1 in sepsis-induced ALI, and unveil potential strategies to treat ALI in clinic.

### Electronic supplementary material

Below is the link to the electronic supplementary material.

Supplementary Material 1

## Data Availability

No datasets were generated or analysed during the current study.
