# Supplementary figures and images for: Crtc1 deficiency protects against sepsis-associated acute lung injury through activating akt signaling pathway
Source: J Inflamm (Lond). 2024 Apr 22;21:12. doi: 10.1186/s12950-024-00385-y (PMC11034098; doi:10.1186/s12950-024-00385-y)

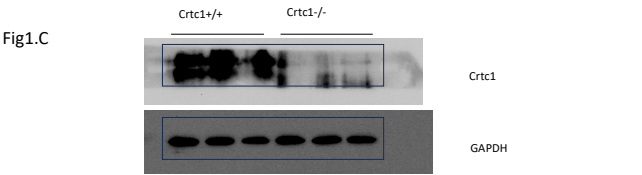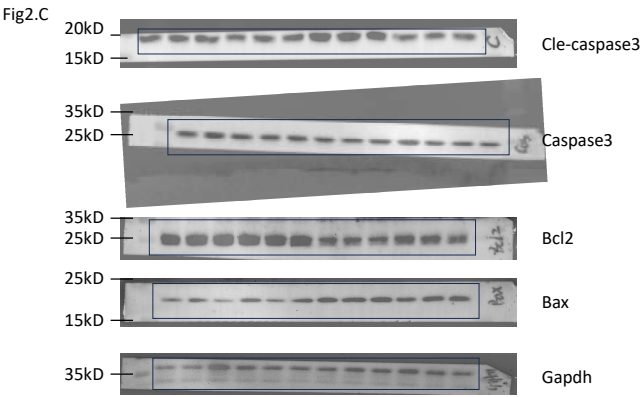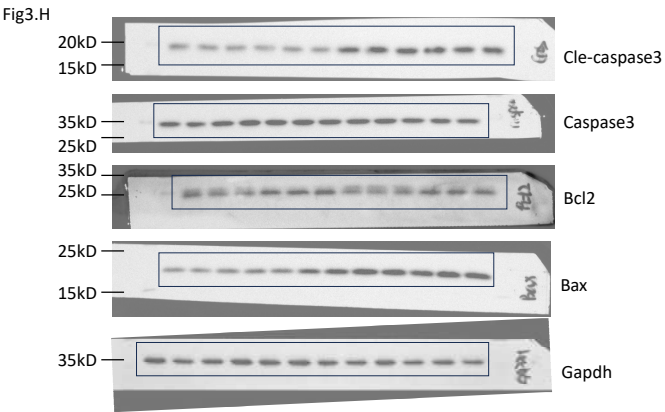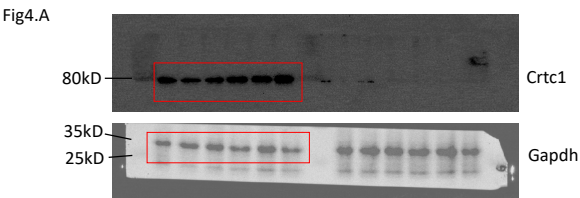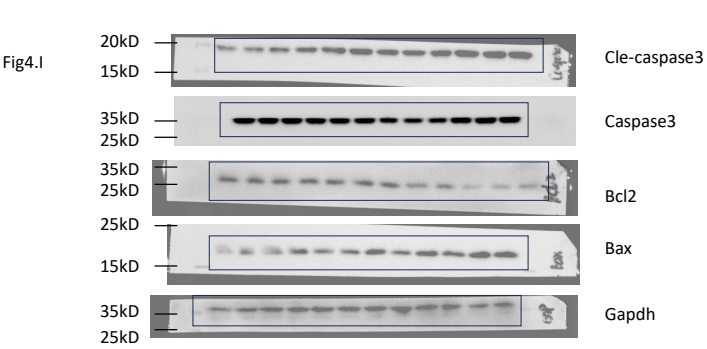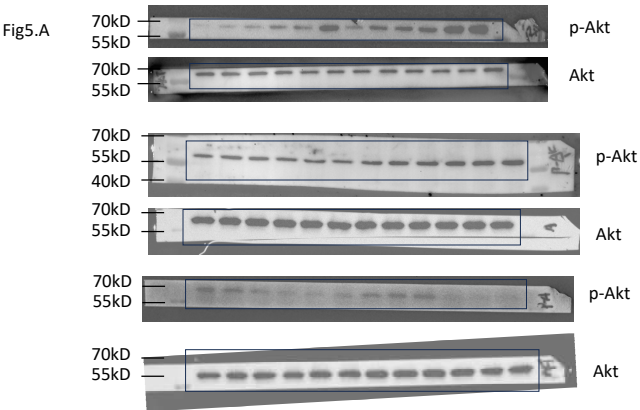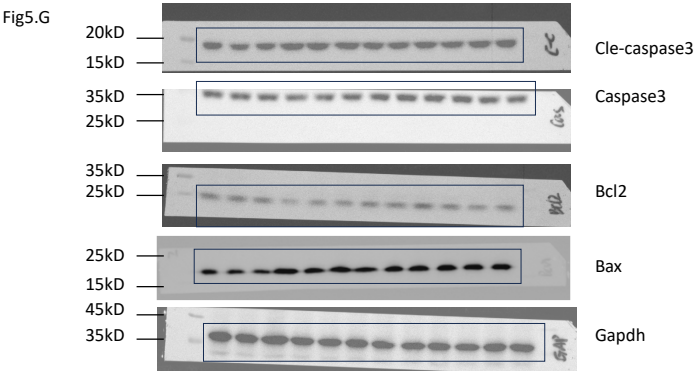

Supplement: Supplementary file 1 — Supplementary Material 1 [file 12950_2024_385_MOESM1_ESM.pdf]
